# To Subscribers

**Published:** 1856-11

**Authors:** 


### EDITORIAL AND MISCELLANEOUS.

### TO SUBSCRIBERS.

We owe our friends an apology for the late appearance of the last number of the Journal; they will, however, readily excuse us, when we inform them that neither of the Editors could give any attention to it—one of them being on his way to Europe, and the other disqualified by sickness. Under these circumstances, the labors connected with the Journal were kindly assumed by an efficient friend, but one who was already overburdened with pressing duties.

Our object will be, hereafter, if possible, to get the number out promptly with the first of the month.

We have been very much gratified of late by public and private assurances, that our labors have not been in vain, and we here return our acknowledgments for the words of commendation and encouragement received from various quarters, and though we have no idea that we deserve the position that some are disposed to give us, we hope yet to be able to furnish a Journal that will be a fit recipient of the compliments which our friends have bestowed.

Our associate, (Prof. W. F. Westmoreland,) as we have already intimated, is now in Europe, and we shall soon expect some account of himself, or rather of what he finds interesting in medicine, across the water. This is his second visit within a few years, and being familiar with medical matters, especially in France, his observations will doubtless be entertaining and profitable.

We would request our contributors to forward their communications in time to reach us, by the first of each month, as it is important that our selections of original matter for the Journal of the succeeding month, should be made during the first week of the preceding. We must again trouble our correspondents with the request, that they will address all letters not immediately connected with the editorial department, to the Treasurer, Dr. J. G. Westmoreland; the Editors have nothing to do with the business of the Journal, and prefer not receiving letters in reference to subscription, distribution to subscribers or advertising, but earnestly invite an extensive and heavy correspondence with collaborators.

